# Lymphangitic Carcinomatosis as a Cause of Malignant Transient Pleural Transudate

**DOI:** 10.1155/2009/598741

**Published:** 2009-09-08

**Authors:** Raquel Garcia Sevila, Encarnacion Barroso, Concepcion Martin, Ignacio Aranda, Santiago Romero

**Affiliations:** ^1^Servicio de Neumologia, Hospital General Universitario de Alicante, 03003 Alicante, Spain; ^2^Servicio de Anatomia Patologica, Hospital General Universitario de Alicante, 03003 Alicante, Spain

## Abstract

Although it is generally accepted that a malignant transient pleural transudate may appear during the early stages of lymphatic obstruction, cases demonstrating such probability are rare in literature. A 67-year-old woman was admitted to hospital because a lymphangitic carcinomatosis and a transudative infrapulmonary pleural effusion with a cytology positive for adenocarcinoma. One month later the effusion keeps being positive for adenocarcinoma but exudative in character. Lymphatic obstruction appears as the cause of the initial transudative characteristics of the pleural effusion.

## 1. Introduction

Although it is generally accepted that a malignant transient pleural transudate may appear during the early stages of lymphatic obstruction [1], cases demonstrating such probability are rare in literature. In fact, as far as we know, the only one case supporting the possibility that a malignancy alone may lead to a transient transudative effusion was published recently by our group [2]. A new similar case, although with some differences that could help to understand some still obscure physiopathological aspects, comes to reaffirm the possible occurrence of this type of transudative effusion.

## 2. Case Report

**Figure 1:**
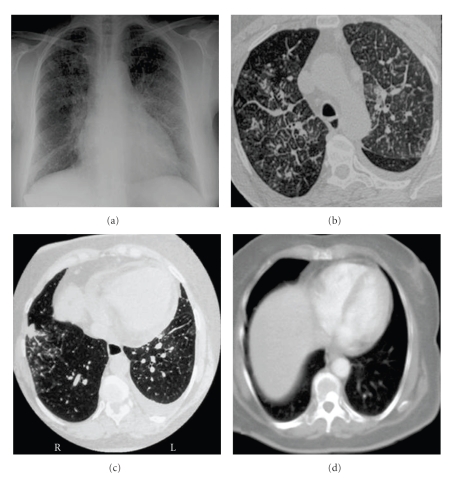
(a) Chest X-ray with interstitial pattern; (b) thoracic high-resolution CT with septal thickening and small left pleural effusion; (c) small nodule in RLL; (d) disappearance of pleural effusion and appearance a vertebral metastasis after 6 cycles of chemotherapy.

**Figure 2:**
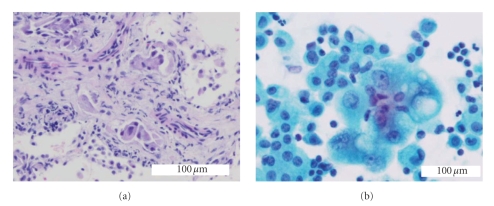
(a) Lymphovascular invasion of lung tissue by adenocarcinoma cells H&E×200; (b) adenocarcinoma cell in transudative pleural effusion. Cell cluster showing variation in nuclear size, prominent nucleoli and vacuolated cytoplasms. Papanicolaou stain×400.

A nonsmoking 67-year-old woman, diagnosed of noninsulin-dependent diabetes mellitus 20 years before, was admitted to the hospital because cough, progressive dyspnea of 4 months of duration, and recently (one week) left pleural chest pain. At admission, she was no febrile, with a heart rate of 74 beats/min and a respiratory rate of 20 breaths/min. Chest auscultation was normal. No evidence of jugular venous distension or hepatomegaly was found. Arterial blood gas (FIO_2_ 0.21) results were pH 7.39; PaCO_2_ 42 mmHg, PaCO_2_ 78 mmHg. Laboratory results were haemoglobin 12.7 g/dL, leukocytes 6660/mm_3_, erythrocyte sedimentation rate 31 mm/h, creatinine 0.7 mg/dL, glucose 141 mg/dL, aspartate aminotransferase 20 U/L, alanine aminotransferase 18 U/L, gamma-glutamyltranspeptidase 22 U/L, lactic dehydrogenase (LDH) 428 U/L, serum carcinoembrionary antigen (CEA) 135 ng/mL. An echocardiography showed a small pericardial effusion and mild pulmonary hypertension without evidence of left ventricular dysfunction. A chest radiograph revealed bilateral pulmonary interstitial pattern and a left infrapulmonary pleural effusion. A thoracic high-resolution-computed tomography scan showed bilateral diffuse thickening of interlobular and perivascular septa compatible with lymphangitic carcinomatosis and a small (2 cm) poorly defined nodule in the right lower lobe (RLL). A small left pleural and pericardial effusions were also evident together with small (1 cm) prevascular and para- tracheal adenopathies (Figure 1). A left thoracentesis yielded light yellow pleural fluid with biochemical characteristics of a transudative effusion that was positive for malignancy on cytological examination. Simultaneous serum and pleural fluid laboratory data are shown in Table 1. An RLL transbronchial lung biopsy showed lymphatic permeation by an adenocarcinoma (Figure 2). A mammography, gastroscopic, and barium enema studies were all negative for malignancy. While looking for a definitive origin of the primitive tumour, that finally was considered to be the RLL pulmonary nodule, the patient did not consent the initiation of the chemotherapy until 1 month later. Pleural fluid obtained by a left thoracentesis the day before the initiation of that therapy was then an exudate (Table 1), while pleural cytology kept being positive for adenocarcinoma cells (Figure 2). The patient was treated with 6 cycles of carboplatin, gencitabine, and docetaxel. After a partial response with disappearance of the pleural effusion, the malignant process progressed, with bone (Figure 1) and brain metastasis and died 11 months after the initiation of the chemotherapy.

## 3. Discussion

Several potential causes could explain the rarity of transudates due to malignant lymphatic obstruction published previously in literature. The short lived period of the transudative features of the effusion, its small size, and the necessity of repeating the diagnostic thoracentesis to demonstrate its changing character, appear as the more probable ones. On the other hand, the presence of a lymphangitic carcinomatosis histologically demonstrated may deter the practice of a diagnostic thoracentesis.

The delay in the transformation from transudate to exudate has been ascribed to the necessity of a finite period (calculated in few weeks) for the protein to accumulate to a level greater than 50% of the serum concentration [1]. Otherwise, it may be that extrapleural lymphatic infiltration allowed the development of a transudative effusion before the tumour invaded the pleural space itself [3]. This last possibility seems the most probable in the published first case [2], in whom within one month, a transudative effusion, with a negative pleural fluid cytology, changed to an exudate, with positive cytology. However, the present case comes to demonstrate that the malignant lymphatic infiltration can produce a pleural effusion with a positive cytology, by an obstructive mechanism, without invading the pleural space itself. This pleural effusion had initially transudative characteristics until, few weeks later, the protein and LDH accumulated in the pleural space due to lymphatic obstruction. The resolution of the effusion simultaneous to that of the lymphangitic carcinomatosis with the chemotherapy points out to an obstructive mechanism.

Most pleural transudates in patients with primary or metastatic pulmonary malignancy are paramalignant that is due to concomitant diseases such as heart or renal failure or secondary to pulmonary atelectasis. Published cases supporting the possibility that a malignancy alone may lead to a transudative effusion in a patient without any other apparent cause for a transudate are infrequent [4, 5]. Assi et al. [4] found that only one of 98 consecutive patients with a positive pleural cytology had a transudate. The exception was a patient with simultaneous congestive heart failure. Afterwards, they concluded that a cytological evaluation for malignant cells of a transudative pleural effusion is not recommended. Moreover, they added that patients with a transudate effusion, even one that is associated with a known malignant tumour, can have the remaining fluid discarded and do not require pleural fluid cytology, as the yield is extremely low [4]. 

 The present case report is an example of an exceptional situation in which invasive techniques (in particular, pleural fluid cytology) used at pleural level may obtain an aetiological diagnosis in patients with pleural transudative effusions. When the cytology is done in a transudative effusion and, the result is positive in absence of alternative causes of transudate, lymphatic obstruction appears as the most probable cause and must be ruled out before proceeding to further pleural invasive explorations.

The Lights criteria have proved to be robust in separating transudates from exudates with a diagnostic accuracy of 96%. However, this case shows that it is less important to know the trans- or exudative nature, but more is the origin/cause of an effusion. We believe that the search for a better marker of pleural fluid should be focused on identifying specific diseases marker and improving clinical management [6].

## Figures and Tables

**Table 1:** Pleural fluid changes between both thoracocenteses.

	16/07/03	07/08/03
Cholesterol mg/dL	26	67
Proteins g/dL PF	1.1	4.1
Proteins g/dL S	7.6	6.8
Proteins PF/S	0.14	0.6
LDH U/L PF	49	461
LDH U/L S	504	514
LDH PF/S	0.1	0.8
Albumin g/dL PF	2.89	2.63
Albumin g/dL S	4.31	3.57
Albumin gradient	1.4	0.9
CEA ng/mL	278	267
Erythrocytes	1760	1480
Cytology	+	+

S: serum; PF: pleural fluid
